# A prototype power assist wheelchair that provides for obstacle detection and avoidance for those with visual impairments

**DOI:** 10.1186/1743-0003-2-30

**Published:** 2005-10-03

**Authors:** Richard Simpson, Edmund LoPresti, Steve Hayashi, Songfeng Guo, Dan Ding, William Ammer, Vinod Sharma, Rory Cooper

**Affiliations:** 1 Department of Rehabilitation Science and Technology; University of Pittsburgh; Pittsburgh, PA, USA; 2 Human Engineering Research Labs; VA Pittsburgh Healthcare System; Pittsburgh, PA, USA; 3 Department of Bioengineering; University of Pittsburgh; Pittsburgh, PA, USA; 4 AT Sciences; Pittsburgh, PA, USA

## Abstract

**Background:**

Almost 10% of all individuals who are legally blind also have a mobility impairment. The majority of these individuals are dependent on others for mobility. The Smart Power Assistance Module (SPAM) for manual wheelchairs is being developed to provide independent mobility for this population.

**Methods:**

A prototype of the SPAM has been developed using Yamaha JWII power assist hubs, sonar and infrared rangefinders, and a microprocessor. The prototype limits the user to moving straight forward, straight backward, or turning in place, and increases the resistance of the wheels based on the proximity of obstacles. The result is haptic feedback to the user regarding the environment surrounding the wheelchair.

**Results:**

The prototype has been evaluated with four blindfolded able-bodied users and one individual who is blind but not mobility impaired. For all individuals, the prototype reduced the number of collisions on a simple navigation task.

**Conclusion:**

The prototype demonstrates the feasibility of providing navigation assistance to manual wheelchair users, but several shortcomings of the system were identified to be addressed in a second generation prototype.

## Background

### Introduction

**Figure 1:**
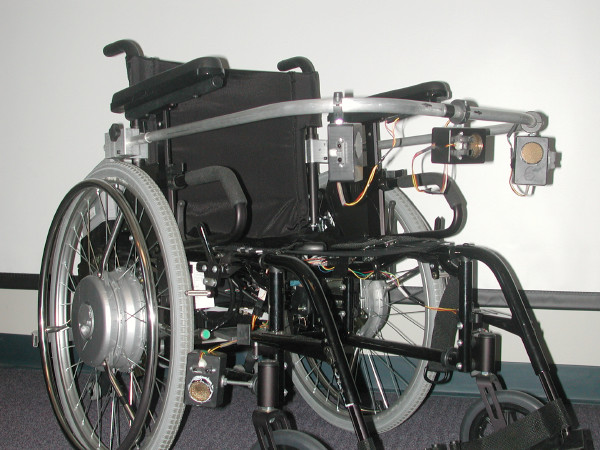
The Smart Power Assistance Module for Manual Wheelchairs (front view).

**Figure 2:**
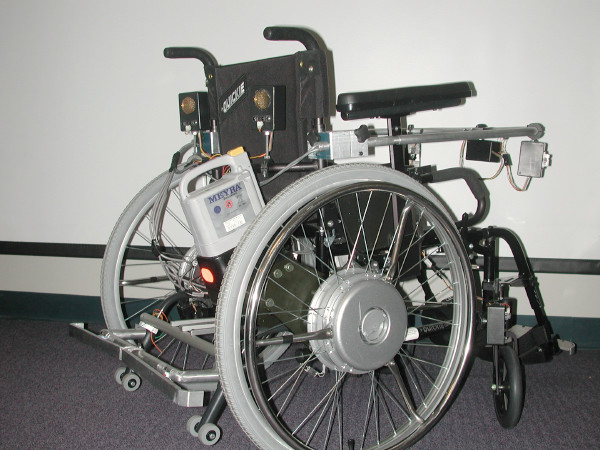
The Smart Power Assistance Module for Manual Wheelchairs (back view).

The concept of *power assistance *for a manual wheelchair is relatively new, and represents a viable alternative for individuals who are unable to generate sufficient propulsion force to use a manual wheelchair, but do not wish to use a traditional powered mobility device [1-3]. In a power assisted manual wheelchair, the traditional rear wheel hubs are replaced with motorized hubs that serve to magnify or reduce (i.e., brake) the propulsive force applied to the rear wheel push rims by the user. Power assistance is being used as the basis for a Smart Power Assistance Module (SPAM) that provides independent power assistance to the right and left rear wheels of a manual wheelchair. The SPAM (shown in Figure 1 and Figure 2) is able to sense the propulsion forces applied by the wheelchair user and provide a smooth ride by compensating for differences in force applied to each wheel. The SPAM is also able to detect obstacles near the wheelchair, and further modify the forces applied to each wheel to avoid obstacles.

The user population for the SPAM consists of individuals with both a visual impairment *and *a mobility impairment that makes it difficult or impossible to ambulate independently using a white cane, guide dog, or other traditional mobility aid for the visually impaired. The American Federation for the Blind (AFB) has estimated that 9.61% of all individuals who are legally blind also use a wheelchair or scooter, and an additional 5.25% of individuals who have serious difficulties seeing (but are not legally blind) also use a wheelchair or scooter (see Appendix). A large number of potential users of the SPAM are expected to be elderly, since visual and physical impairments often accompany the natural aging process. In 2000, approximately 13% of the total US population, or an estimated 35 million people, were age 65 or older; with about 2% at least age 85. By 2030, the older population is projected to double, expanding to 70 million. People age 85 and older are the fastest growing segment of the American population and the US Census Bureau estimates that there are now 65,000 centenarians [4].

### Relevant Research

Currently, the majority of non-ambulatory visually-impaired individuals are seated in a manual wheelchair and pushed by another person [5]. Depending on the extent of useful vision remaining, individuals with low-vision can operate an unmodified manual wheelchair, powered wheelchair or scooter, but the risk of an accident obviously increases with increased visual impairment. There are reports of individuals using a white cane [6] or guide dog [7] along with a wheelchair, but this is not common practice.

Despite a long history of research in smart power wheelchairs, there are very few smart wheelchairs currently on the market. Two North American companies, Applied AI and ActivMedia, sell smart power wheelchair prototypes for use by researchers, but neither system is intended for use outside of a research lab. The CALL Center of the University of Edinburgh, Scotland, has developed a wheelchair with bump sensors, a single sonar sensor, and the ability to follow tape tracks on the floor for use within a wheeled-mobility training program [8]. The CALL Center smart power wheelchair is sold in the United Kingdom (UK) and Europe by Smile Rehab, Ltd. (Berkshire, UK) as the "Smart Wheelchair." The "Smart Box," which is also sold by Smile Rehab in the UK and Europe, is compatible with wheelchairs using either Penny and Giles or Dynamics control electronics and includes bump sensors (but not sonar sensors) and the ability to follow tape tracks on the floor.

One common feature of all of these smart wheelchairs is that they are based on power wheelchairs. Power wheelchairs are a convenient platform for researchers, but have several disadvantages when compared with manual wheelchairs. In general, manual wheelchairs are lighter and more maneuverable than power wheelchairs, and can be transported in a car. Manual wheelchairs that make use of power assist hubs are heavier than traditional manual wheelchairs, and can be more difficult to disassemble for transport depending on how the hubs are attached to the frames, but still provide many of the advantages of traditional manual wheelchairs.

In a search of the literature, only one other smart wheelchair was identified that was based on a manual wheelchair. The Collaborative Wheelchair Assistant [9] controls the direction of a manual wheelchair with small motorized wheels that are placed in contact with the wheelchair's rear tires to transfer torque. Unlike the SPAM, however, the Collaborative Wheelchair Assistant restricts the wheelchair's travel to software-defined "paths."

One of the few products that is commercially-available and accommodates a manual wheelchair is the Wheelchair Pathfinder [10], a commercial product sold by Nurion Industries that can be attached to a manual or power wheelchair. The Wheelchair Pathfinder uses sonar sensors to identify obstacles to the right, left or front of the wheelchair and a laser range finder to detect drop-offs in front of the wheelchair. Feedback is provided to the user through vibrations or differently-pitched beeps. The Wheelchair Pathfinder differs from the SPAM in that the Wheelchair Pathfinder has limited sensor coverage and cannot alter the speed or direction of travel of the wheelchair to avoid obstacles.

## Methods

**Figure 3:**
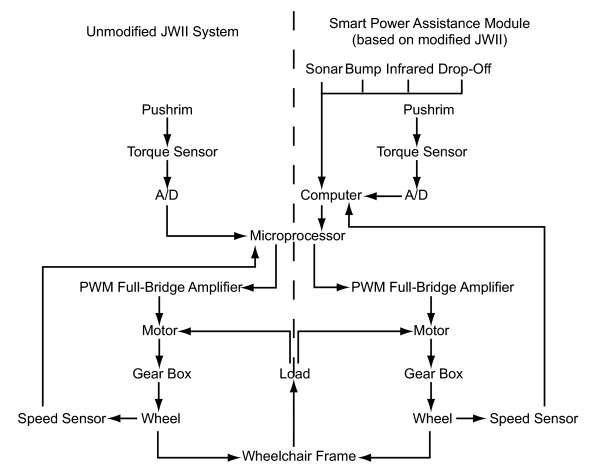
Schematic for unmodified JWII system (left) and SPAM (right).

The right side of Figure 3 shows the design of the SPAM prototype, which has been implemented "on top of" a pair of Yamaha JWII power-assist pushrim hubs (sold in the United States as the Quickie Xtender). The SPAM is able to sense (1) the propulsive force applied to each rear wheel of the wheelchair, (2) the magnitude and velocity of rotation of each rear wheel, and (3) the location of obstacles relative to the wheelchair. Information from all sensors is collected by a microprocessor which integrates information about the user's input and the surrounding environment, and passes command signals to the JWII system's microprocessor.

**Figure 4:**
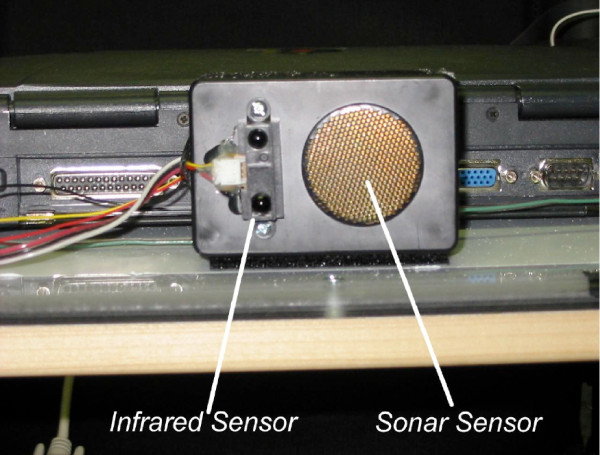
Sensor Module.

**Figure 5:**
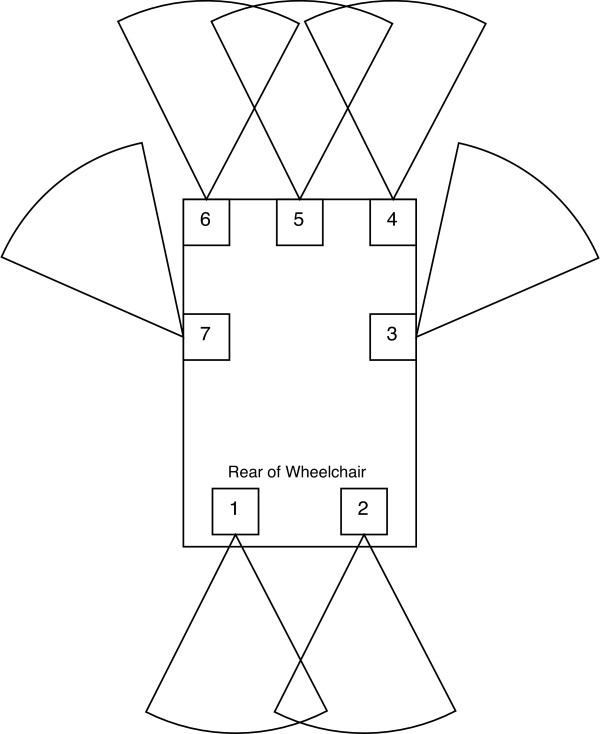
Position of Sensors on SPAM.

Several types of sensors have been integrated into the SPAM. These sensors are used for (1) tracking the state of the wheelchair (e.g., wheel velocity, torque applied to each rear wheel by the user) and (2) locating obstacles in the wheelchair's environment. Obstacles are identified using infrared rangefinders, sonar sensors and bump sensors. The sonar sensors have a maximum range of 3.05 m and a minimum range of 2.54 cm. The advantages of a smaller range are that (1) the frequency of sonar readings is increased and (2) the sonar system is able to detect obstacles that are extremely close to the wheelchair, which is important for passing through doorways. Infrared range finders provide a focused, highly modulated infrared beam, providing absolute ranging based on simple triangulation. The result is an accurate range value between 0.1 and 1.0 meters in a variety of circumstances. The infrared signal functions at extremely steep angles, even exceeding sixty degrees, and does so both indoors and outdoors, even in bright sunlight. The infrared rangefinders and sonar sensors are housed in. 09 m × .06 m × .04 m boxes (shown in Figure 4), which are referred to as "sensor modules." Seven sensor modules are mounted on the current prototype. Bump sensors are attached to both footrests and the "anti-tippers" of the manual wheelchair, and are implemented using simple contact switches placed behind mechanical levers. Figure 5 shows how the sensor modules were positioned on the SPAM.

The SPAM's control software shares control of the wheelchair with the wheelchair operator. The wheelchair operator is responsible for choosing when – and in which direction – the wheelchair moves, while the SPAM modifies the speed of the wheelchair based on the proximity of obstacles in the wheelchair's current direction of travel. The algorithm currently employed by the SPAM forces the rear wheels to turn either at exactly the same speed and direction (moving the wheelchair straight forward or straight backward) or at the same speed and opposite directions (rotating the wheelchair in place). This greatly simplifies the task of avoiding obstacles but limits the wheelchair user's flexibility in choosing paths of travel.

The navigation assistance software was written in C and runs on a TattleTale™ (manufactured by Onset Technologies) 8-bit microprocessor. User input (either forward, backward or turn in place) and sensor data are combined into "cases" that are used to make obstacle avoidance decisions. The specific cases that are in use at any one time varies depending on the specific behavior that is desired from the SPAM (e.g., passing through a narrow doorway versus driving quickly through a room with few obstacles). No single case can cause the software to prevent both forward/backward movement and turning, but multiple cases can be triggered at once and result in a situation in which the wheelchair will not move in any direction. The motorized hubs can be turned off in these situations, at which point the SPAM behaves like a normal (but heavy) manual wheelchair.

## Results

**Figure 6:**
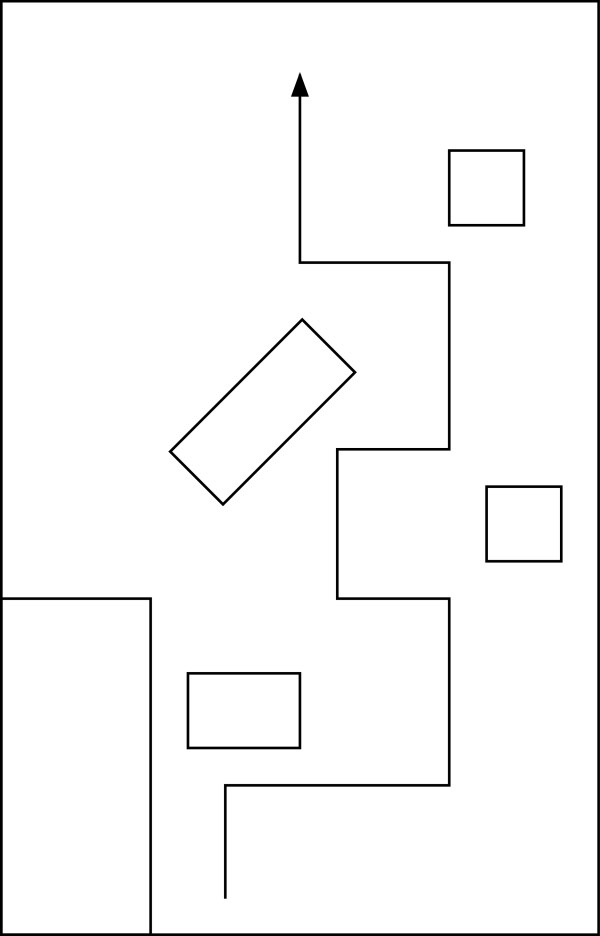
Obstacle Course 1.

**Figure 7:**
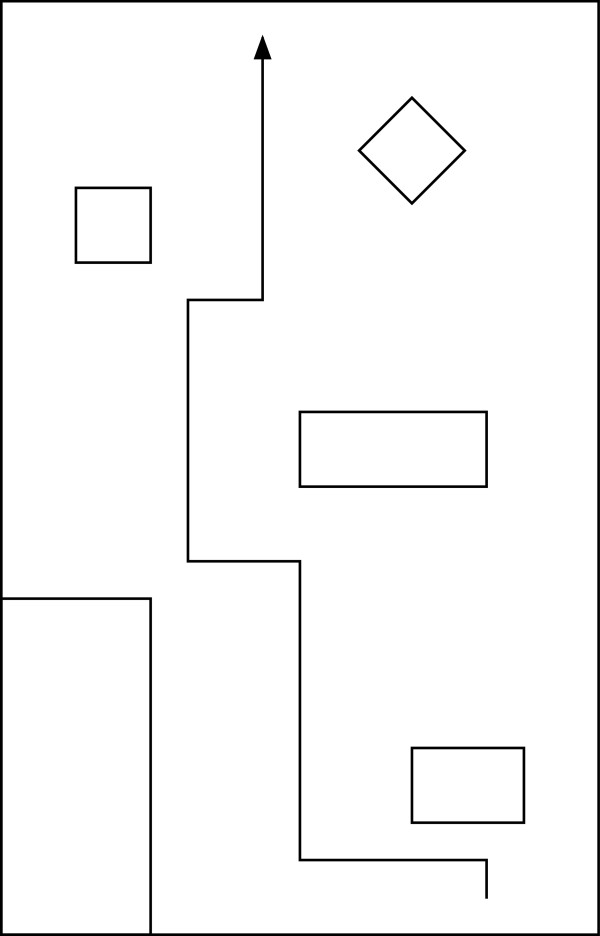
Obstacle Course 2.

Four able-bodied members of the investigative team and an individual who is blind, but does not have a mobility impairment, took part in an evaluation of the SPAM prototype. Approval for this research was obtained from the University of Pittsburgh's Institutional Review Board. All participants used the SPAM to complete the two obstacle courses shown in Figure 6 and Figure 7. Able-bodied participants were asked to complete each course three times blindfolded with navigation assistance from the SPAM. The participant who is blind completed each course nine times, in alternating sets of three trials. The sets of three trials alternated between the SPAM providing navigation assistance (condition woa) and the SPAM acting as a normal manual wheelchair (i.e., the hubs were powered but the SPAM was not acting to avoid collisions; condition noa). All subjects completed trials with Course 1 first.

**Figure 8:**
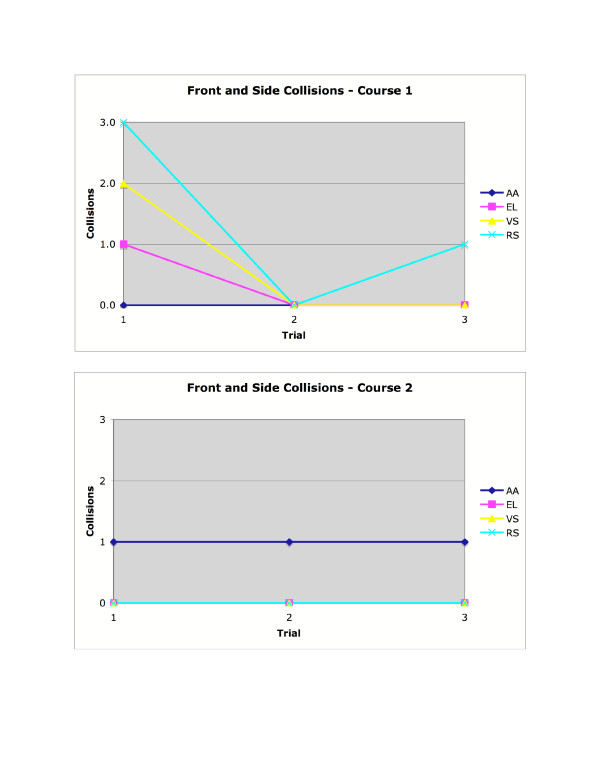
Collisions for able-bodied participants, in courses 1 and 2.

**Figure 9:**
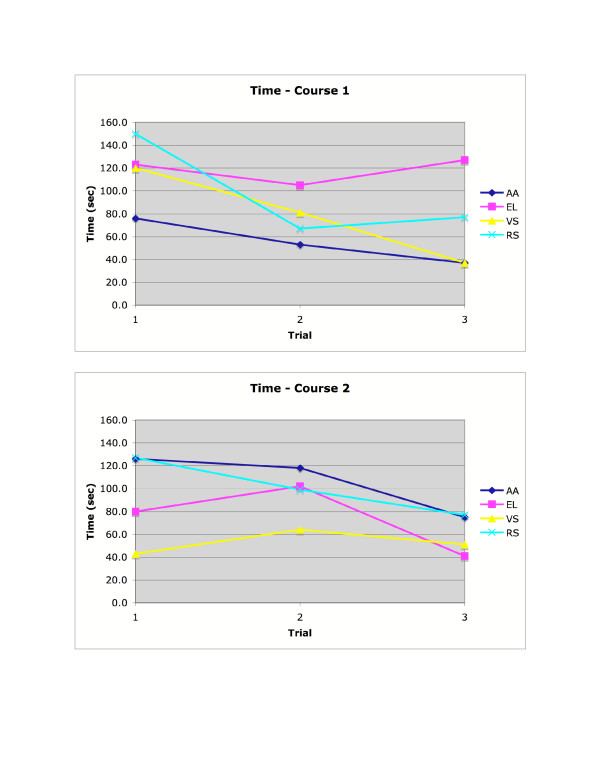
Time to complete the navigation task for able-bodied participants, in courses 1 and 2.

As shown in Figure 8, the SPAM did not completely eliminate collisions for able-bodied subjects. However, three of four subjects had no collisions after the first trial on Course 1, and only one of the four subjects had a collision in any trial on Course 2. As shown in Figure 9, able-bodied subjects generally completed both navigation tasks more quickly by the third trial.

**Figure 10:**
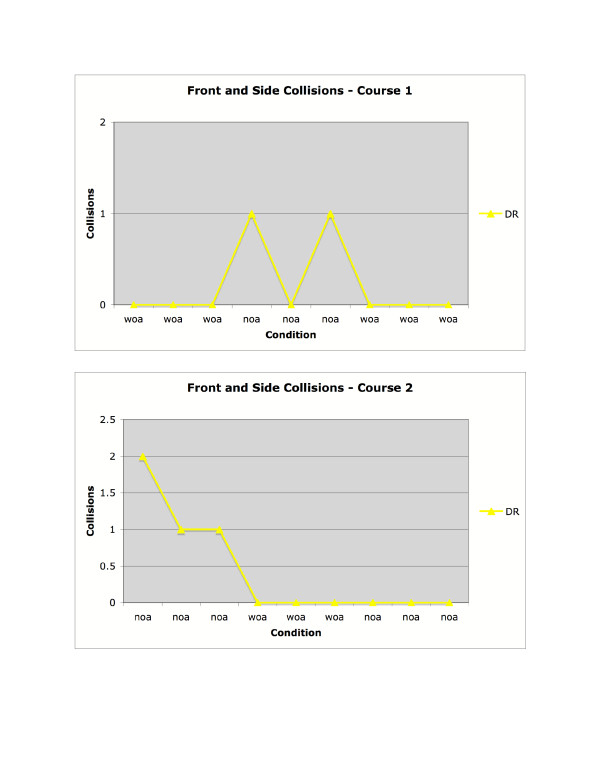
Collisions for the visually-impaired participant, in courses 1 and 2.

**Figure 11:**
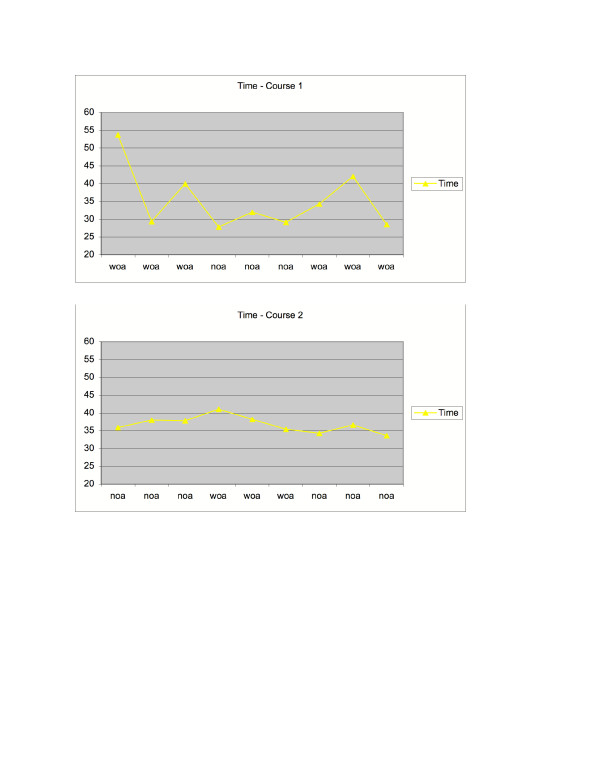
Time to complete the navigation task for the visually-impaired participant, in courses 1 and 2.

As shown in Figure 10, the subject who was visually-impaired had no collisions in the first three trials on Course 1 (with obstacle avoidance active) but did have collisions on Course 1 when obstacle avoidance was removed. On Course 2, where obstacle avoidance was not active during the first three trials, the visually-impaired subject had collisions in the first three trials but did not have collisions once obstacle avoidance was introduced. As shown in Figure 11, there was not a consistent effect of experimental condition on time in Course 1. In Course 2, time to complete the task was extremely consistent despite experimental condition.

## Discussion

One clear observation from our preliminary evaluations of the SPAM is the distinct difference between able-bodied, but blindfolded, individuals and individuals who are completely blind. The participant who is blind was much better at localizing the sound target and keeping track of his location in the course than any of the able-bodied participants. The blind participant also found it much easier to learn the layout of the course. One possible implication of these results is that the SPAM may be more useful for individuals who are newly visually impaired. Another possible implication is that the SPAM may be very useful in novel or frequently-changing environments, but not particularly useful in well-known, static environments.

Our preliminary evaluation of the SPAM demonstrates that the SPAM can increase the safety of visually-impaired manual wheelchair users. Of course, there is a large difference between a constrained laboratory environment and real-world environments, and much additional development and testing remains to be done. Our evaluation also identified several shortcomings. In particular, navigation assistance increased the time required to complete the navigation task. This was the result of an overly conservative obstacle avoidance algorithm, which slowed the SPAM more than necessary.

Our ability to control the SPAM was limited by our decision to retain the original electronics of the JWII hubs in place. This greatly simplified the development process, and allowed us to quickly produce a prototype that could be tested. The trade-off, however, was that our microprocessor and control software were not communicating directly with the motors within the hubs but were, instead, communicating with the JWII microprocessor and control software which controlled the motors. The control algorithms built into the JWII acted as a filter that made small adjustments in the speed and direction of the wheelchair difficult. This is why the motion of the SPAM was limited to straight forward, straight backward, and turning in place.

One unanticipated benefit of using power assist hubs which emerged during development was the ability to provide "haptic feedback" to the wheelchair user. As the SPAM approaches an obstacle, the hubs provide greater resistance. This allows the user to get an impression of the environment around the wheelchair through a series of forward pushes and rotations in place. In addition to individuals with visual impairments, this haptic feedback may also prove helpful for people with traumatic brain injuries.

## Conclusion

The lessons learned from the first SPAM prototype are being incorporated into a second generation SPAM prototype (currently under development). Most importantly, the microprocessor used by the JWII hubs is being replaced with a new (programmable) microprocessor, which will allow the SPAM to provide much smoother and more nuanced control of the wheelchair. New enclosures have also been designed for the sensors that provide increased mounting flexibility, and have increased the number of modules. The additional sensor modules have forced us to abandon the case-based approach to obstacle avoidance, and alternative algorithms are being pursued.

## Declaration of competing interests

AT Sciences has applied for a patent for the SPAM. AT Sciences will be submitting a Phase II SBIR proposal to the National Eye Institute based, in part, on the results contained in this manuscript. Dr. Simpson is not employed by, nor does he hold any stocks or shares in, AT Sciences. Dr. Simpson participated in this research through a subcontract negotiated between AT Sciences and the University of Pittsburgh.

## Authors' contributions

RS, EL and RC conceived of the project and participated in its design and coordination. RS implemented the obstacle avoidance software, conducted the user trials and drafted the manuscript. SG, DD, SH and WA implemented the hardware for the SPAM, and interfaced the TattleTale microprocessor with the JWII electronics. VS reconstructed the SPAM to complete additional user testing. All authors read and approved the final manuscript.

## Appendix

The American Federation for the Blind performed the following analysis using data from the 1994 and 1995 National Health Interview Survey on Disability, Phase I. Analysis used variables for being legally blind (location 422) and having serious difficulty seeing (location 401), using a manual wheelchair (location 524), an electric wheelchair (location 526), or a scooter (location 528), and age recode 2 (location 30). Data were extracted from phase I person files for each year, the design variables were recoded, the 2 years of data were combined, and the final weights were adjusted (wfta/2) in order to compute crosstabs in SUDAAN. Among all persons who are legally blind(1), 9.61% (se = 1.10) use a wheelchair. The 95% CI for this statistic is 7.41% to 11.81%. Among persons who are legally blind, (see Table 2) and 7.79% of individuals under the age of 65 (see Table 3), use a wheelchair.

**Table 1:** Use of Mobility Aids – All Ages

	Legally Blind	Serious Difficulty Seeing but not legally blind	US Population
Totals	1057389.5	5315541	259994178
Uses Any Kind of Wheelchair (Manual, Electric or Scooter)	101565	279070.5	1668244.5
Percent	9.61	5.25	0.64

**Table 2:** Use of Mobility Aids – Ages 65 and Over

	Legally Blind	Serious Difficulty Seeing but not legally blind	US Population
Totals	482935	2541294	31156585
Uses Any Kind of Wheelchair (Manual, Electric or Scooter)	56789	181005	924301
Percent	11.76	7.12	2.97

**Table 3:** Use of Mobility Aids – Under Age 65

	Legally Blind	Serious Difficulty Seeing but not legally blind	US Population
Totals	574454	2774247	228837592
Uses Any Kind of Wheelchair (Manual, Electric or Scooter)	44776	98065	743943
Percent	7.79	3.53	0.33
